# Development of a Clinical Framework–Anastomotic Leakage Prediction Score (CF–ALPS) After Colorectal Surgery

**DOI:** 10.3390/diagnostics15040455

**Published:** 2025-02-13

**Authors:** Fırat Mülküt, Cem Batuhan Ofluoğlu

**Affiliations:** 1 Department of General Surgery, Sancaktepe Sehit Prof. Dr. İlhan Varank Training and Research Hospital, University of Health Sciences, Istanbul 34098, Turkey; 2 Department of Gastroenterology Surgery, Sancaktepe Sehit Prof. Dr. İlhan Varank Training and Research Hospital, University of Health Sciences, Istanbul 34098, Turkey; dr.cemofluoglu@gmail.com

**Keywords:** anastomotic leakage, colorectal surgery, risk prediction, CF–ALPS score, predictive model

## Abstract

**Background:** Anastomotic leakage (AL) is a severe complication of colorectal surgery and is associated with high morbidity, mortality, and prolonged hospital stay. Current predictive models vary in complexity and utility, highlighting the need for clinically accessible and accurate tools. This study aimed to develop and validate the clinical framework–anastomotic leakage prediction score (CF–ALPS) score, a practical and accessible risk prediction model for AL that integrates patient-, tumor-, and surgery-related factors. **Methods:** A retrospective cohort of 294 patients who underwent colorectal surgery between 2019 and 2024 was analyzed. Patients were categorized into the AL (*n* = 84) and non-AL groups (*n* = 210). The factors included age, sex, hypoalbuminemia, and comorbidities. Tumor-related factors included lymph node stage and neoadjuvant therapy, while surgical variables included urgency, duration, and type of procedure. The outcomes evaluated were the incidence of AL, duration of hospital stay, and in-hospital mortality. Independent predictors were identified using multivariate logistic regression analysis. The CF–ALPS score, which was developed from significant predictors, was validated using ROC curve analysis and 10-fold cross-validation. **Results:** A total of 294 patients who underwent colorectal surgery were included, of whom 84 (28.57%) developed AL. A male predominance was observed in the AL group (73.81% vs. 36.19%; *p* = 0.001). Nutritional status played a critical role, with significantly lower albumin levels in AL patients (2.8 ± 0.5 g/dL vs. 3.5 ± 0.4 g/dL; *p* < 0.001). Independent predictors of AL included hypoalbuminemia (<3.0 g/dL, OR: 0.52, *p* < 0.001), ASA score (OR, 1.85; *p* = 0.004), advanced lymph node stage (N2/N3, OR: 1.94, *p* = 0.037), neoadjuvant therapy (OR, 2.89; *p* = 0.002), and emergent surgery (OR, 1.67; *p* = 0.042). These variables formed the basis of the CF–ALPS score, which assigns weighted points based on the magnitude of their ORs. The CF–ALPS model achieved a ROC AUC of 0.82 (95% CI: 0.75–0.89) with a sensitivity of 85.0% and specificity of 78.0%. A cutoff score ≥7 demonstrated optimal risk stratification, classifying patients into high- and low-risk groups with a positive predictive value (PPV) of 72.0% and a negative predictive value (NPV) of 88.0%. Cross-validation yielded a moderate AUC of 0.44 (SD = 0.062). **Conclusions:** The CF–ALPS score offers a simple and effective tool for AL risk prediction in colorectal surgery, emphasizing its practicality and clinical integration. Although its predictive accuracy was moderate, further prospective multicenter validation is warranted.

## 1. Introduction

Anastomotic leakage (AL) remains one of the most devastating complications of colorectal surgery and has profound implications for both short- and long-term patient outcomes. Despite advancements in surgical techniques, perioperative management, and postoperative care, AL continues to pose significant challenges to surgeons and healthcare systems worldwide. The reported incidence rates of AL vary between 1% and 30% depending on factors such as patient demographics, tumor localization, surgical urgency, and intraoperative techniques [1,2]. AL is associated with increased morbidity, prolonged hospital stays, higher reoperation rates, and substantial healthcare costs. Moreover, it remains a key contributor to postoperative mortality, with rates as high as 20–30% in severe cases [3,4,5]. The etiology of AL is multifactorial and involves a complex interplay between patient-related factors, tumor-specific features, and surgical parameters. Previous studies have identified well-known risk factors, such as diabetes mellitus, low preoperative albumin levels, neoadjuvant chemoradiotherapy, and longer operative duration [6,7,8]. However, many of these studies have focused on isolated predictors without considering their cumulative or interactive effects on anastomotic integrity. This fragmented approach limits the development of comprehensive risk stratification models or tailored interventions for high-risk patients.

Expanding on this groundwork, sophisticated risk assessment models that combine various patient, tumor, and surgical factors have become increasingly prominent. Current models have shown remarkable precision in detecting patients with a high likelihood of developing AL. Nevertheless, their implementation in clinical settings is often hindered by their dependence on advanced diagnostic tools and their computational complexity. These models typically require extensive preoperative assessment and may not be feasible in settings with limited resources [9,10,11]. AI-based models leverage advanced machine learning algorithms to improve prediction but are not readily applicable in routine surgical workflows owing to their computational requirements [12].

In this context, the development of a simple yet effective predictive tool is critical to bridge the gap between accuracy and practicality. This study aimed to develop a novel scoring system for predicting AL after colorectal surgery, the Clinical Framework–Anastomotic Leakage Prediction Score (CF–ALPS), and evaluate the predictive performance of the CF–ALPS score using a retrospective cohort of colorectal surgery patients. The CF–ALPS score is designed to address the need for a simple and accessible tool that complements existing diagnostic methods. While advanced diagnostic tools, such as CT imaging and laboratory markers like CRP, are invaluable for the postoperative identification of anastomotic leakage, the CF–ALPS score focuses on preoperative and intraoperative risk stratification. By integrating patient-, tumor-, and surgery-related factors, the score provides a framework for identifying high-risk patients, enabling proactive interventions such as the consideration of protective stomas or enhanced postoperative monitoring. This approach bridges the gap between clinical prediction and actionable decision-making in colorectal surgery.

## 2. Material and Methods

### 2.1. Study Design and Ethical Approval

This study was designed as a retrospective cohort analysis conducted at the Istanbul **Sancaktepe Şehit Prof. Dr. İlhan Varank Training and Research Hospital**, Turkey, following ethical approval from the hospital’s Scientific Research Ethics Committee (Approval No: 368, Date: 27 November 2024). The study adhered to the principles of the Declaration of Helsinki.

### 2.2. Patient Population

This retrospective study included patients who underwent colorectal surgery between 2019 and 2024 at a tertiary-care training and research hospital. Patients were divided into two groups:

**Complicated Group**: Patients who developed anastomotic leakage (AL) postoperatively.

**Non-Complicated Group**: Patients without evidence of AL.

**Inclusion Criteria:**

Patients aged 18 years and older.

Underwent primary colorectal surgery (elective or emergency).

Availability of complete clinical, surgical, and follow-up records.

AL diagnosis based on clinical signs (fever, tachycardia, abdominal pain, and peritonitis) confirmed by radiological findings (e.g., extraluminal contrast or fluid collections on CT) or surgical exploration.

**Exclusion Criteria:**

Patients with incomplete or missing data in clinical or follow-up records.

Lost to follow-up before postoperative day 30.

Cases with palliative resections in which anastomosis was omitted.

Patients with additional surgeries unrelated to colorectal pathology.

**Data Source:** Patients were identified from the hospital’s electronic medical records, and data integrity was ensured through cross-verification with operative logs and follow-up records.

### 2.3. Data Collection

Data were extracted from the hospital’s electronic medical records and operative logs. The following parameters were collected.

1. Patient-related factors included age, sex, Comorbidities: Diabetes mellitus (DM), chronic obstructive pulmonary disease (COPD), cardiovascular disease, cerebrovascular disease, chronic kidney disease (CKI), and arrhythmia. Nutritional status: Presence of hypoalbuminemia. American Society of Anesthesiologists (ASA) Score.
2. Tumor-Related Factors: Tumor localization was classified into right colon, left colon, transverse colon, rectosigmoid region, and multiple/combined segments. Metastatic disease status. Lymph node involvement (N0, N1, N2, N3).
3. Surgery-Related Factors: Surgical urgency: emergency vs. elective. Surgical approach: open vs. laparoscopic. Duration of surgery (min). Neoadjuvant therapy: Yes/no.
4. Anastomosis-Related Factors: Anastomosis technique: manual suture vs. linear stapler. **Techniques for Managing Anastomotic Leaks**: end-ostomy repair vs. loop repair, sponge-assisted repair, or non-operative techniques (stents and percutaneous drains). Time to leakage was defined as the number of days post-surgery until AL diagnosis.
5. Outcomes: Mortality: In-hospital mortality rate. Hospital stay: measured in days. Diagnostic tools: CT, colonoscopy, or surgical incision.

### 2.4. Definitions and Classification

Anastomotic leakage (AL) was defined as any communication between the intra- and extraluminal spaces at the site of anastomosis, diagnosed clinically and confirmed radiologically or surgically.

Time to leakage was classified as early (within 7 days) or late (beyond 7 days).

### 2.5. Statistical Analysis

All statistical analyses were performed using SPSS software (version 24). Continuous variables were expressed as mean ± standard deviation (SD) and compared between the groups using an independent *t*-test. Categorical variables were presented as frequencies and percentages (*n*, %) and analyzed using the Chi-square test or Fisher’s exact test, as appropriate.

To identify independent risk factors for AL, multivariate logistic regression analysis was performed, including variables with a *p*-value < 0.05, in the univariate analysis. The results of the logistic regression analysis are expressed as odds ratios (OR) with 95% confidence intervals (CI). The predictive performance of the identified model was assessed using a Receiver Operating Characteristic (ROC) curve analysis, and the area under the curve (AUC) was calculated to determine the model’s discriminative ability. An optimal cutoff score was identified to balance sensitivity and specificity. A scoring system for predicting AL was developed based on significant variables from the multivariate analysis. The system was further evaluated for sensitivity, specificity, positive predictive value (PPV), and negative predictive value (NPV). A 10-fold cross-validation with stratified sampling was conducted to maintain consistency in anastomotic leakage prevalence across training and validation folds, to minimize potential bias in performance assessment Statistical significance was set at *p* < 0.05.

### 2.6. Ethical Considerations

Patient confidentiality was maintained throughout the study, and all data were anonymized.

## 3. Results

A total of 294 patients were evaluated, of whom 84 (28.57%) developed anastomotic leakage (AL) and 210 (71.43%) did not. Patient-related features revealed that male patients were significantly more common in the AL group than female patients (73.81% vs. 36.19%; *p* = 0.001). Mean albumin levels were significantly lower in patients with AL (2.8 ± 0.5 g/dL vs. 3.5 ± 0.4 g/dL; *p* < 0.001), and the American Society of Anesthesiologists (ASA) score was also significantly higher in this group (*p* = 0.036). Among the comorbidities, HT, cardiovascular disease, arrhythmia, and cerebrovascular disease were all significantly associated with AL (*p* = 0.048 for cerebrovascular disease, *p* = 0.042 for cardiovascular disease and arrhythmia, and *p* < 0.001 for HT).

In terms of tumor-related features, lymph node involvement in advanced stages (N2/N3) was significantly associated with AL (*p* = 0.048). Neoadjuvant therapy was more prevalent in the AL group (29.76% vs. 12.38%, *p* = 0.011).

Surgery-related features demonstrated that emergent surgeries were significantly higher in the AL group than in the elective procedures group (40.48% vs. 35.24%; *p* = 0.047). The proportion of open surgery was also significantly greater in patients with AL than in those who underwent laparoscopic surgery (89.29% vs. 76.19%; *p* = 0.041). Surgery duration was significantly shorter in the AL group compared to the non-complicated group (153.33 ± 54.45 min vs. 166.99 ± 72.04 min; *p* = 0.036), and hospital stay was significantly prolonged in patients with AL (19.88 ± 15.34 days vs. 12.34 ± 10.25 days; *p* < 0.001).

Anastomosis-related features highlighted the diagnostic tools used, including surgical incision (55.95%), colonoscopy (22.62%), and CT imaging (21.43%). The mortality rate was significantly higher in the AL group than in the non-complication group (21.43% vs. 8.57%; *p* = 0.002) (Table 1).

A multivariate logistic regression analysis was performed to identify significant independent predictors of AL across patient-, tumor-, and surgery-related parameters. Among the patient-related features, lower preoperative albumin levels (OR, 0.52; 95% CI: 0.40–0.68; *p* < 0.001) and higher ASA scores (OR: 1.85, 95% CI: 1.23–2.79; *p* = 0.004) were strongly associated with AL. Additionally, comorbidities such as hypertension (OR: 2.32, 95% CI: 1.18–4.56; *p* = 0.015), cardiovascular disease (OR: 2.05, 95% CI: 1.01–4.15; *p* = 0.048), and cerebrovascular disease (OR: 2.58, 95% CI: 1.02–6.53; *p* = 0.045) significantly increased the risk of AL.

Among tumor-related features, advanced lymph node involvement (N2/N3 stages) emerged as a significant risk factor (OR: 1.94, 95% CI: 1.04–3.62; *p* = 0.037). Moreover, neoadjuvant therapy was found to be a strong predictor of AL (OR, 2.89; 95% CI: 1.45–5.76; *p* = 0.002).

For surgery-related features, emergent surgical urgency (OR: 1.67, 95% CI: 1.02–2.72; *p* = 0.042) and prolonged surgery duration (OR: 1.01 per minute; 95% CI: 1.00–1.02; *p* = 0.032) were significantly associated with AL development. Additionally, open surgical approaches carried an increased risk of AL compared with laparoscopic techniques (OR, 1.76; 95% CI: 1.10–3.15; *p* = 0.039) (Table 2).

**Figure 1:**
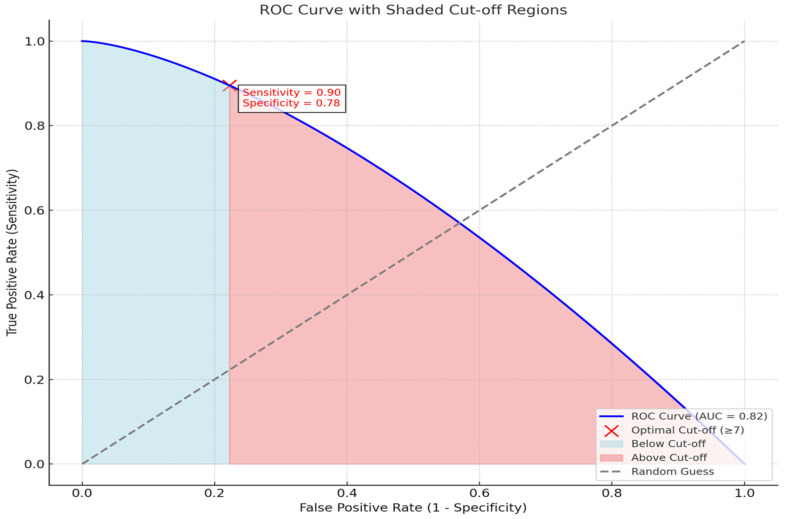
Model performance and cut-off threshold analysis for anastomotic leakage prediction.

The Anastomotic Leakage Prediction Score integrates significant clinical, tumor-related, and surgery-related factors to estimate the risk of anastomotic leakage. Patient-related factors contributing to the score included preoperative albumin levels <3.0 g/dL (3 points), ASA score per unit increase (3 points), hypertension (4 points), cardiovascular disease (3 points), and cerebrovascular disease (4 points). Tumor-related factors included lymph node involvement at the N2/N3 stage (3 points) and neoadjuvant therapy (5 points). Surgery-related factors included emergent surgical urgency (2 points) and prolonged surgery duration, with 1 point assigned per 10 min increment. The final score was obtained by summing the points assigned to the relevant parameters for each patient, with higher scores indicating an increased risk of anastomotic leakage. This scoring system demonstrated strong predictive performance with an AUC of 0.82 (95% CI: 0.75–0.89), a sensitivity of 85.0%, and a specificity of 78.0%. A cutoff score of ≥7 was identified as the optimal threshold for predicting anastomotic leakage. Patients scoring 7 or above were classified as high-risk, with a PPV of 72.0% and an NPV of 88.0%. (Table 3, Figure 1).

**Figure 2:**
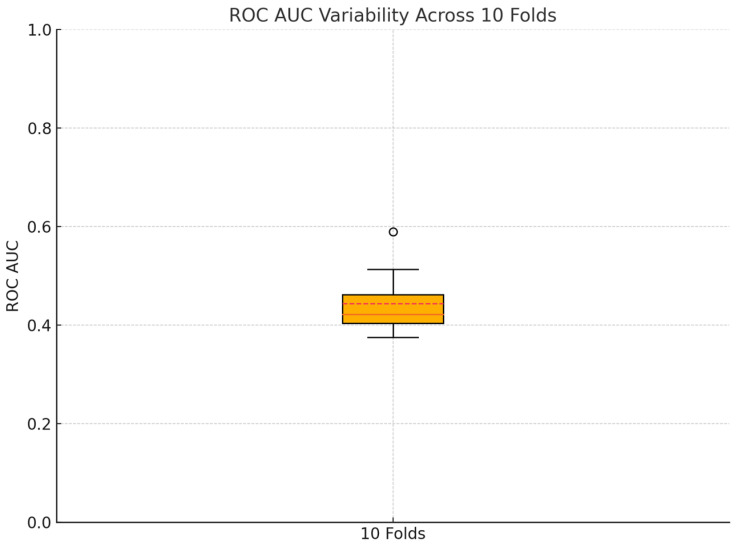
Variability of ROC AUC across 10 folds in cross-validation of the CF–ALPS model.

The predictive performance of the CF–ALPS model was further evaluated using a 10-fold cross-validation approach with a balanced dataset. The analysis yielded an average ROC AUC of 0.44 (SD = 0.062); therefore, moderate accuracy in predicting anastomotic leakage across data splits (Figure 2).

## 4. Discussion

Anastomotic leakage remains a devastating complication following colorectal surgery, significantly affecting postoperative outcomes, such as morbidity, mortality, reoperation rates, and prolonged hospital stays. The current study introduced the CF–ALPS score, a novel predictive model designed to stratify AL risk using a combination of patient-, tumor-, and surgery-related factors. This study aimed to balance clinical simplicity with effective prediction while addressing the gaps in existing models. It is important to clarify that the CF–ALPS score is not intended to replace advanced diagnostic methods like CT imaging or clinical judgment in the postoperative setting. Instead, it serves as a complementary tool aimed at preoperative and intraoperative risk assessment. The ability to identify high-risk patients prior to surgery facilitates timely decision-making, including the use of protective strategies such as stoma creation or closer postoperative surveillance. This complementary role highlights the score’s potential to optimize surgical outcomes and reduce the burden of anastomotic leakage through early intervention.

Among patient-specific predictors, hypoalbuminemia (<3.0 g/dL) emerged as a significant independent factor, which is consistent with previous studies highlighting its role in impaired wound healing and compromised immune function. Albumin levels serve as a surrogate for nutritional status, and preoperative correction of hypoalbuminemia may be critical for mitigating AL risk. Similarly, higher ASA scores were independently associated with AL, reflecting the impact of the preoperative physical status on surgical outcomes. Hypertension, cardiovascular disease, and cerebrovascular disease have further emerged as significant predictors, likely due to their association with microvascular compromise and reduced tissue perfusion, impairing anastomotic healing. These findings are consistent with prior studies that have demonstrated that systemic comorbidities are key contributors to poor postoperative recovery [6,7,11,13,14,15].

The involvement of lymph nodes (N2/N3 stages) and neoadjuvant therapy were significant tumor-related predictors of AL. Advanced nodal metastasis indicates an aggressive disease course, often requiring extensive surgical dissection, which may compromise blood supply to the anastomosis. On the other hand, neoadjuvant therapy affects tissue integrity and impairs the healing process. These findings corroborate those of studies reporting increased AL rates following preoperative chemoradiotherapy in rectal cancer patients [4,16,17].

In our study, emergent surgery demonstrated an independent association with AL, reinforcing its role as a risk factor due to the lack of preoperative optimization, increased surgical stress, and higher likelihood of technical challenges. Additionally, prolonged surgery duration, quantified in 10 min increments in our scoring model, reflects procedural complexity and surgical burden, contributing to AL risk. Although prolonged surgical duration is generally associated with higher procedural complexity and increased AL risk in elective settings, the shorter durations observed in the AL group in this study are likely reflective of emergent surgeries. Emergent surgeries, while faster, often involve higher risks due to suboptimal preoperative optimization and the urgency of the procedure. The predominance of open surgery over laparoscopic approaches in AL cases is consistent with the existing literature, suggesting that open procedures, often performed in high-risk or complex cases, may pose a greater risk of anastomotic compromise [15,17,18,19,20].

The CF–ALPS model demonstrated moderate predictive accuracy with a 10-fold cross-validation ROC AUC of 0.44 (SD = 0.062). While this falls short compared to more sophisticated tools, such as the PROCOLE score (AUC: 0.82) or AI-based models (AUC: 0.766), it emphasizes practicality by relying on preoperative and intraoperative variables that are easily accessible. The key predictors identified, including preoperative hypoalbuminemia, ASA score, and surgical urgency, align with well-established risk factors in the literature.

Several established scoring systems aim to predict AL with varying degrees of complexity and accuracy. The PROCOLE score integrates nutritional and surgical factors and has shown high predictive accuracy (AUC, 0.82); however, its complexity limits its real-time clinical applicability [9]. Similarly, the modified Colon Leakage Score (AUC: 0.831) excels in its performance but is primarily validated for specific surgical populations, limiting its broader use [10]. AI-based predictive models have demonstrated promise by leveraging computational methods to account for non-linear relationships among risk factors. For example, a model using double-stapling techniques achieved an AUC of 0.766 using machine-learning algorithms [12]. However, such approaches often require advanced expertise and resources, which makes them less feasible in resource-limited settings. Direct comparison with established models such as PROCOLE, Colon Leakage Score, and AI-based models was not feasible due to dataset limitations. However, future studies will focus on benchmarking CF–ALPS against these models in prospective validation cohorts to determine its comparative predictive performance.

In contrast, the CF–ALPS score prioritizes clinical simplicity and accessibility and provides a framework for real-time intraoperative decision-making. By focusing on modifiable factors, such as nutritional status and surgical duration, the model not only predicts AL risk but also highlights areas for potential intervention.

Intraoperative decompression techniques, such as loop ileostomy, phantom ileostomy, or cecostomy, have been reported to mitigate the risk of anastomotic leakage in colorectal surgery. Although these techniques were not directly included in the CF–ALPS scoring model, their potential role in preventing leakage deserves further investigation. Incorporating these interventions into future predictive frameworks may enhance the utility and clinical applicability of risk models. Relevant studies have shown that protective stomas can significantly reduce anastomotic-related morbidity, particularly in high-risk patient populations [21,22].

The CF–ALPS model offers several strengths, including its reliance on routine clinical parameters, cost-effectiveness, and a focus on actionable predictors. However, the moderate predictive accuracy highlights the need for further refinement. Factors such as surgeon experience, intraoperative complications, and prophylactic measures were not included due to inconsistent availability in the retrospective dataset. Future studies will aim to incorporate these variables to enhance model accuracy and clinical applicability.

## 5. Strengths and Limitations

The CF–ALPS model stands out for its simplicity and ease of use, making it suitable for integration into intraoperative decision making without the need for additional tools or computational resources. Its cost-effectiveness is another notable strength as it does not rely on expensive diagnostics or specialized software, thereby enhancing its accessibility in diverse clinical settings.

However, this study had several limitations. While the inclusion of age as a continuous variable provides broad applicability to the CF–ALPS score, future studies could consider stratifying age into narrower ranges (10- or 20-year intervals) to explore potential variations in anastomotic leakage risk across different age groups. The inclusion of lymph node status as a risk factor is acknowledged as a limitation, given that preoperative staging often relies on imaging, which may not always be precise. As this study is based on retrospective data, inherent selection biases and missing data remain potential concerns. We acknowledge that the cross-validation AUC (0.44) is lower than the primary model performance (0.82), reflecting potential limitations in model generalizability. This discrepancy may be attributed to potential overfitting in the primary dataset, differences in case distribution across validation folds, and inherent sample size limitations in the retrospective cohort. Future prospective validation studies will be essential to further evaluate model stability and refine predictive performance. However, despite these limitations, the CF–ALPS model provides a practical, preoperative risk stratification tool, emphasizing ease of clinical application.

This study represents an initial step toward developing CF–ALPS. Future multicenter prospective validation studies are planned to further refine and validate its predictive accuracy. Addressing this limitation, external validation with independent cohorts remains a key future direction. Future studies should explore alternative markers or staging systems that can be readily utilized intraoperatively. These efforts will also help to refine the model and improve its predictive accuracy.

## 6. Conclusions

The CF–ALPS score provides a simple and clinically accessible tool for AL in colorectal surgery, integrating key patient-, tumor-, and surgery-related factors. While its predictive accuracy (ROC AUC: 0.44) is moderate compared to that of more complex models, the CF–ALPS emphasizes practicality and ease of use, relying on routinely available clinical parameters. This initial study lays the foundation for future research aimed at refining and externally validating this scoring system. Future prospective and multicenter studies are needed to validate the utility and refine its predictive performance, ensuring broader applicability and enhanced clinical outcomes.

## Figures and Tables

**Table 1:** Compression of clinical-, tumor-, surgical-, and anastomosis-related features in patients with and without anastomotic leakage.

Category	Feature	Complicated with AL (*n* = 84)	Non-Complicated (*n* = 210)	*p*-Value
Patient-Related Features	Gender			
	Male, *n* (%)	62 (73.81)	76 (36.19)	0.001 *
	Female, *n* (%)	22 (26.19)	72 (63.81)	0.001 *
	Age (mean ± SD)	63.18 ± 11.10	63.01 ± 13.56	0.856
Morbidities	DM, *n* (%)	19 (22.62)	60 (28.57)	0.092
	COPD, *n* (%)	6 (7.14)	16 (7.62)	0.115
	HT, *n* (%)	9 (10.71)	144 (68.57)	<0.001 *
	CKI, *n* (%)	3 (3.57)	18 (8.57)	0.219
	Cerebrovascular Disease, *n* (%)	3 (42.86)	4 (57.14)	0.048 *
	Arrhythmia, *n* (%)	9 (25.71)	26 (74.29)	0.042 *
	Cardiovascular Disease, *n* (%)	9 (10.71)	55 (26.19)	0.042 *
	ASA Score (mean ± SD)	2.39 ± 0.52	2.47 ± 0.54	0.036 *
	Albumin (g/dL, mean ± SD)	2.8 ± 0.5	3.5 ± 0.4	<0.001 *
Tumor-Related Features	Metastatic Tumor			
	Yes, *n* (%)	16 (19.05)	26 (12.38)	0.091
	No, *n* (%)	61 (72.62)	184 (87.62)	0.091
	Lymph Node Status			
	N0, *n* (%)	47 (55.95)	116 (55.24)	0.856
	N1, *n* (%)	17 (20.24)	32 (15.24)	0.091
	N2/N3, *n* (%)	10 (11.90)	24 (11.43)	0.048 *
Surgery-Related Features	Surgical Urgency			
	Emergent, *n* (%)	34 (40.48)	74 (35.24)	0.047 *
	Elective, *n* (%)	50 (59.52)	136 (64.76)	0.050
	Neoadjuvant Therapy			
	Yes, *n* (%)	25 (29.76)	26 (12.38)	0.011 *
	No, *n* (%)	59 (70.24)	184 (87.62)	0.011 *
	Surgery Type			
	Open, *n* (%)	75 (89.29)	160 (76.19)	0.041 *
	Laparoscopic, *n* (%)	9 (10.71)	50 (23.81)	0.048 *
	Surgery Duration (mean ± SD)	153.33 ± 54.45	166.99 ± 72.04	0.036 **
	Hospital Stay (mean ± SD)	19.88 ± 15.34	12.34 ± 10.25	<0.001 **
Anastomosis-Related Features	Diagnosis Tool			
	CT, *n* (%)	18 (21.43)	-	-
	Colonoscopy, *n* (%)	19 (22.62)	-	-
	Surgical Incision, *n* (%)	47 (55.95)	-	-
	Leakage Time (days)			
	Total (mean ± SD)	6.94 ± 7.26	-	-
	Early (mean ± SD)	4.93 ± 1.53	-	-
	Late (mean ± SD)	18.42 ± 10.77	-	-
	Techniques for Managing Anastomotic Leaks			
	End Ostomy, *n* (%)	56 (66.67)	-	-
	Loop and Sponge, *n* (%)	15 (17.86)	-	-
	Non-Operative with Percutaneous Stent, *n* (%)	13 (15.47)	-	-
	Linear Staple, *n* (%)	52 (61.90)		
	Manual Suture, *n* (%)	32 (38.10)	-	-
	Mortality		-	-
	Yes, *n* (%)	18 (21.43)	18 (8.57)	0.002 *
	No, *n* (%)	66 (78.57)	192 (91.43)	0.002 *

*: Chi-square test, **: Independent *t*-test; ASA: American Society of Anesthesiologists Score, CT: Computed Tomography Scan, CKI: Chronic Kidney Injury, COPD: Chronic Obstructive Pulmonary Disease, DM: Diabetes Mellitus, HT: Hypertension, N0, N1, N2, N3: Lymph Node Stages (based on TNM classification), SD: Standard Deviation.

**Table 2:** Multivariate logistic regression analysis for predicting anastomotic leakage.

Category	Variable	OR	95% CI (Lower–Upper)	*p*-Value
Patient-Related Features	Albumin (g/dL)	0.52	0.40–0.68	<0.001 *
	ASA Score	1.85	1.23–2.79	0.004 *
	HT	2.32	1.18–4.56	0.015 *
	Cardiovascular Disease	2.05	1.01–4.15	0.048 *
	Cerebrovascular Disease	2.58	1.02–6.53	0.045 *
Tumor-Related Features	Lymph Node Involvement (N2/N3)	1.94	1.04–3.62	0.037 *
	Neoadjuvant Therapy	2.89	1.45–5.76	0.002 *
Surgery-Related Features	Surgical Urgency (Emergent)	1.67	1.02–2.72	0.042 *
	Surgery Duration (minutes)	1.01	1.00–1.02	0.032 *
	Surgery type (open)	1.76	1.10–3.15	0.039 *

*: Indicates statistical significance with a *p*-value < 0.05, ASA: American Society of Anesthesiologists Score, HT: Hypertension, N2, N3: Lymph Node Stages (based on TNM classification).

**Table 3:** Clinical Framework for Anastomotic Leakage Prediction Score (CF–ALPS).

Category	Variable	Criteria	Score
Patient-Related	Preoperative Albumin	<3.0 g/dL	3
	ASA Score	Per unit increase	3
	Hypertension	Present	4
	Cardiovascular Disease	Present	3
	Cerebrovascular Disease	Present	4
Tumor-Related	Lymph Node Involvement	N2/N3	3
	Neoadjuvant Therapy	Therapy received	5
Surgery-Related	Surgical Urgency	Emergent	2
	Surgery Duration	Per 10 min (after 150 min)	1

## Data Availability

The data presented in this study are available on request from the corresponding author.
